# Psychosocial, health and demographic characteristics of quality of life among patients with acute myeloid leukemia and malignant lymphoma who underwent autologous hematopoietic stem cell transplantation

**DOI:** 10.1590/S1516-31802007000600012

**Published:** 2007-11-01

**Authors:** Ladislav Slovacek, Birgita Slovackova, Ladislav Jebavy, Zuzana Macingova

**Affiliations:** Second Department of Internal Medicine, Charles University Hospital, Hradec Kralove, Czech Republic

**Keywords:** Quality of life, Patients, Leukemia, Lymphoma, Bone marrow transplantation, Qualidade de vida, Pacientes, Leucemia, Linfoma, Transplante de medula óssea

## Abstract

**CONTEXT AND OBJECTIVE:**

This study evaluated the effect of selected psychosocial, health and demographic characteristics of quality of life (QOL) among patients treated with autologous hematopoietic stem cell transplantation (HSCT).

**DESIGN AND SETTING:**

This was a retrospective study at Charles University Hospital, Hradec Kralove.

**METHODS:**

The Czech version of the international generic European Quality-of-Life questionnaire (EQ-5D) was applied to evaluate QOL among patients with acute myeloid leukemia (AML) and malignant Hodgkin's and non-Hodgkin's lymphoma (ML). The total number of respondents was 36: 12 with AML (seven males and five females) and 24 with ML (11 males and 13 females). The mean age of AML respondents was 46 years and the mean age of ML respondents was 44.5 years.

**RESULTS:**

Age, smoking status and education level had statistically significant effects on QOL among AML respondents (p < 0.05), and age had a statistically significant effect on QOL among ML respondents (p < 0.05). The overall QOL among AML and ML respondents was generally good: the mean EQ-5D score among AML respondents was 71.5% and among ML respondents it was 82.7%.

**CONCLUSION:**

The QOL among AML and ML respondents treated with autologous HSCT was good. However, patients more than 50 years old, smokers and patients with lower education levels presented worse QOL. These findings need to be better evaluated in longitudinal studies, using large samples.

## RESUMEN

**CONTEXTO E OBJETIVO:**

O transplante de células tronco hematopoiéticas (TCTH) é um método a ser utilizado na terapêutica das doenças hemato-oncológicas e tumores sólidos, assim como no tratamento de doenças não-malignas. O TCTH tem influência na evolução da doença e na qualidade de vida do doente, assim como outros métodos curativos.

**TIPO ESTUDO E LOCAL:**

Este estudo é retrospectivo. Foi desenvolvido no Departamento Clínico de Hematologia do 2^o^ Departamento de Medicina Interna do Hospital da Universidade de Charles em Hradec Kralove, República Checa.

**MÉTODOS:**

Amostra de 36 adultos sujeitos ao tratamento com transplante autólogo de células tronco do progenitor, sendo 12 com leucemia mielóide aguda: sete homens e cinco mulheres, e 24 com linfoma maligno: 11 homens e 13 mulheres. Foi utilizada a versão checa do questionário internacional de qualidade de vida européia versão EQ – 5D.

**RESULTADOS:**

Houve associação estastiticamente significante entre qualidade de vida e idade, fumantes, pacientes com baixo nível educacional em ambos os sujeitos com leucemia mielóide aguda (p < 0.05). Houve associacão estatisticamente significante entre qualidade de vida e idade em ambos os sujeitos com linfoma maligno (p < 0.05).

**CONCLUSÃO:**

A qualidade de vida dos pacientes com linfoma maligno e leucemia mielóide aguda, submetidos a transplante autólogo do progenitor, mantém-se preservada na maioria dos pacientes. Pacientes com idade acima de 50 anos, baixo nível educacional e fumantes têm pior qualidade de vida. Estes fatores devem ser melhor avaliados em estudos longitudinais e com amostras maiores.

## INTRODUCTION

Quality of life (QOL) is defined as patients’ subjective evaluation of their life situation.^1,2^ QOL ratings contain information on individuals’ physical, psychological, social and spiritual condition.^3,4^ QOL evaluations are carried out by means of generic and specific questionnaires.^1-4^

Hematopoietic stem cell transplantation (HSCT) is a specific therapeutic method used for biomodulatory antitumor therapy on hematological malignancies and solid tumors. It is also used for therapy on non-tumor and hereditary diseases.^1^

HSCT influences the further course of the disease and through this it influences patients’ QOL in the same way as do other therapeutic methods.^1,2^

## OBJECTIVE

To assess the relationship between patients’ characteristics (psychosocial: education level, marital status and religion; health: number of associated diseases, smoking status, type of disease and time elapsed since HSCT; demographics: age and sex) and QOL among patients with acute myeloid leukemia (AML) and malignant Hodgkin's and non-Hodgkin's lymphoma (ML) who were treated with autologous HSCT.

## MATERIAL AND METHODS

We carried out this retrospective study among AML and ML patients who had been treated with autologous HSCT between 2001 and 2003. This study was based on data obtained between September 1, 2004, and January 31, 2005. We evaluated the effect of selected psychosocial, health and demographic characteristics of these AML and ML patients on their QOL. The total number of respondents was 36. All respondents were aged over 18 years. This study was approved by Charles University Hospital Ethics Committee.

The evaluation of QOL among these respondents was performed using the Czech version of the international generic European Quality-of-Life questionnaire (EQ-5D).^1,2,5^ This questionnaire evaluates two indicators: one objective and the other subjective. The objective indicator includes five dimensions of QOL: ability to move, self-sufficiency, usual activity, pain/complaints and anxiety/depression. Three responses that express the degree of complaints are offered for each question (no complaints, mild complaints or severe complaints). The outcome from this is the EQ-5D score (QOL dimension), which has values from 0 to 1 (0 = worst health condition; 1 = best health condition). The subjective indicator consists of a visual analog scale (0 = worst health condition; 100 = best health condition). The outcome from this is the EQ-5D visual analog scale (VAS) (subjective health condition), which has values from 0 to 100. The questionnaires were evaluated by means of descriptive analysis in accordance with the European Quality-of-Life Group methodology.^1,2,5^

The statistical analysis used analysis of variance (ANOVA). Descriptive analysis was used to evaluate the QOL questionnaire. The statistical analysis was conducted using the StatSoft Statistica database, version 7.1. p-values < 0.05 were considered significant.

## RESULTS

The total number of respondents was 36 (18 males and 18 females). The mean age of all the respondents was 46 years (age range: 18-72). The number of AML respondents was 12 (7 males and 5 females). The mean age of the AML respondents was 46 years (age range: 27-68). The number of ML respondents was 24 (11 males and 13 females). The mean age of the ML respondents was 44.5 years (age range: 18-72). The number of respondents with Hodgkin's lymphoma was nine and with non-Hodgkin's lymphoma was 15.

**Graph 1:**
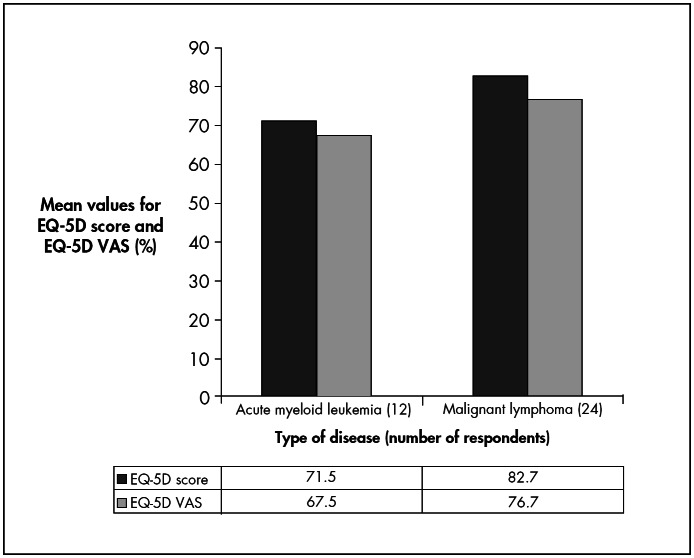
Dependence of European Quality-of-Life questionnaire (EQ-5D) score and European Quality-of-Life questionnaire (EQ-5D) VAS on type of disease among respondents who underwent autologous hematopoietic stem cell transplantation (HSCT) between 2001 and 2003 (n = 36, p < 0.05).

The overall QOL among AML and ML respondents (Graph 1) was generally good. The mean EQ-5D score among AML respondents was 71.5% and among ML respondents it was 82.7%, while the mean EQ-5D VAS among AML respondents was 67.5% and among ML respondents it was 76.7%).

Age, smoking status and education level had statistically significant effects on QOL among AML respondents (p < 0.05) and age had a statistically significant effect on QOL among ML respondents (p < 0.05) (Tables 1 and 2). The results showed that the QOL among AML and ML respondents declined with increasing age. The AML respondents with secondary or university levels of education had higher QOL than did the AML respondents with primary or apprentice levels of education. The AML respondents who were smokers had lower QOL than did the non-smoking AML respondents.

**Table 1:** Comparison between mean European Quality-of-Life questionnaire (EQ-5D) score and mean European Quality-of-Life questionnaire (EQ-5D) visual analog scale (VAS) for different age groups, smoking status and education levels among respondents with acute myeloid leukemia (AML) who underwent autologous hematopoietic stem cell transplantation (HSCT) between 2001 and 2003 (n = 12, p < 0.05)

		Number of respondents	Mean value for EQ-5D score (%)	Standard deviation	Mean value for EQ-5D VAS (%)	Standard deviation
**Age range**	20-29	1	70	0	60	0
	30-39	1	98	0	95	0
	40-49	5	86.2	15.7	73.6	13.9
	50-59	3	60	14.5	58.3	2.4
	60-69	2	61	15	56	4
**Smoking status**	Non-smokers	6	90.7	11.4	77.2	16.4
	Smokers	6	59.5	14.1	57.8	3.5
**Education level**	Elementary	2	43	4.2	53.5	2.12
	Apprentice	3	67	8.5	60	0
	Secondary	3	74	4.0	65	8.7
	University	4	98	0	82	17.9

**Table 2:** Comparison between mean European Quality-of-Life questionnaire (EQ-5D) score and European Quality-of-Life questionnaire (EQ-5D) visual analog scale (VAS) for different age groups among respondents with malignant Hodgkin's and non-Hodgkin's lymphoma (ML) who underwent autologous hematopoietic stem cell transplantation (HSCT) between 2001 and 2003 (n = 24, p < 0.05)

		Number of respondents	Mean value for EQ-5D score (%)	Standard deviation	Mean value for EQ-5D VAS (%)	Standard deviation
**Age range**	20-29	3	98	0	83.3	0
	30-39	6	98	0	86.7	0
	40-49	4	98	0	78.8	14.5
	50-59	7	67.3	14.5	73	2.4
	60-69	4	64	15	65	4

## DISCUSSION

This study showed that selected psychosocial, health and demographics characteristics of AML and ML patients treated with autologous HSCT had significant effects on their QOL. Increasing age and increasing numbers of associated diseases were correlated with lower QOL. This was probably due to over­all fatigue, emotional difficulties, lower over­all physical fitness and worse quality of sleep.

This explanatory study is the first investigation of QOL among AML and ML patients who had been treated with autologous HSCT in the Czech Republic, and it is one of the few studies carried out in countries of the former Eastern European Block. It has shown some patient characteristics that are associated with better QOL, and thus how they influence post-HSCT QOL. Through this, AML and ML patients who are more than 50 years old, are smokers and have low education levels can be better observed by their doctors, with the aim of developing better strategies to improve their QOL. Further studies will be needed with longitudinal data.

We are also aware that our study may be limited by certain other factors. Firstly, this study deals only with the effect of these selected psychosocial, health and demographic characteristics on overall QOL. We could also have studied some other characteristics. But we decided to investigate these characteristics because the patients were able and willing to provide this information retrospectively in an anonymous investigation. Secondly, in this study we used the generic European Quality-of-Life questionnaire (EQ-5D) to evaluate QOL among our patients. We decided to use this because our patients were only able and willing to complete this questionnaire. Our patients emphasized that this questionnaire was very intelligible and especially brief. We originally wanted to use the Czech version of the Functional Assessment of Cancer Therapy-General (FACT-G) questionnaire or the European Organization for Research and Treatment of Cancer Quality of Life Core Questionnaire version 3 (EORTC QLQ-C30) questionnaire, but our patients had a negative reaction in relation to completing one of these questionnaires. The patients emphasized that these two questionnaires were very comprehensive and demanded too much of their time.

## CONCLUSION

The AML and ML respondents treated with autologous HSCT presented good QOL. Patients who were more than 50 years old, smokers and individuals with lower education levels presented worse QOL. These characteristics need to be better evaluated in longitudinal studies, using large samples.
